# Estimation of SPAD values in litchi based on improved LSTM with fusion of IoT and multispectral image texture features

**DOI:** 10.3389/fpls.2026.1858723

**Published:** 2026-06-05

**Authors:** Zhen Li, Xiaoyi Chen, Junxing He, Leyuan Chen, Peng Gao

**Affiliations:** 1 College of Artificial Intelligence and Low-Altitude Technology, South China Agricultural University, Guangzhou, China; 2 Division of Citrus Machinery, China Agriculture Research System of Ministry of Agriculture and Rural Affairs (MARA), Guangzhou, China

**Keywords:** IoT, LSTM, precision agriculture, SPAD, texture feature

## Abstract

Litchi is an important economic fruit in southern China, and its precision management relies on the rapid and accurate estimation of the Soil and Plant Analyzer Development (SPAD) values in leaves. Addressing the limitations of existing SPAD detection methods, such as limited rapid coverage, inadequate modeling of dynamic environmental interference, and shallow fusion of multi-source data, this study constructed an Internet of Things (IoT) system to collect real-time environmental data from a litchi orchard, combined with unmanned aerial vehicle (UAV) multispectral imagery to obtain canopy vegetation index and texture features. A Long Short-Term Memory (LSTM) network model integrated with a feature level attention mechanism (MLSTM) was proposed to fuse IoT time-series data, vegetation index, and high dimensional texture features for dynamic SPAD value prediction. The results indicate that multi-source feature fusion significantly improves SPAD estimation accuracy. The MLSTM model achieved optimal performance under the all-features situation, with a coefficient of determination (R²) of 0.897 and a root mean square error (RMSE) of 2.638, outperforming other comparative models. The attention mechanism effectively enhanced the model’s focus on key features, improving feature utilization efficiency and model interpretability. The multi-source data fusion method and MLSTM model proposed in this study enable high precision, dynamic estimation of SPAD values in litchi leaves, providing reliable data support for precision fertilization, stress diagnosis, and yield prediction in litchi orchards, as well as theoretical support for promoting the practical application of this technology in smart agriculture.

## 1 Introduction

Litchi chinensis (use “litchi” thereafter) is an important specialty economic fruit in southern China, with extensive cultivation areas, especially in tropical and subtropical regions such as Guangdong, Guangxi, Fujian, and Hainan, where it has formed a large scale industrial pattern. According to relevant statistics, China’s litchi cultivation area consistently remains at 333,500 hectares, with an annual production exceeding 2 billion kilograms. It plays a critical role in ensuring farmers’ income growth, promoting regional economic development, and enriching the supply of agricultural products in the market (Yin et al., 2024). However, as the industry continues to develop, cultivation models relying on traditional experience and extensive management exhibit certain shortcomings in terms of precision, efficiency, and coping with labor shortages, which have become significant constraints to the further high level development of the litchi industry. Smart cultivation management has therefore become a central strategy for addressing these challenges. For smart litchi orchard management, the core lies in utilizing modern information technologies such as the Internet of Things (IoT), Artificial Intelligence (AI), and remote sensing to achieve whole process, real-time, and dynamic perception as well as precise regulation of the orchard environment and tree physiological states. Achieving smart management requires precise control and understanding of key physiological and biochemical indicators in crops (Qiao et al., 2024). Among numerous physiological indicators, chlorophyll is widely regarded as a core indicator for assessing plant health status, nutritional levels, and photosynthetic efficiency due to its close correlation with plant photosynthetic capacity, nitrogen nutrition status, growth stress levels, and ultimately fruit quality and yield (Yakushev et al., 2022). At the same time, in litchi orchards, rapid and accurate estimation of Soil and Plant Analyzer Development (SPAD) values in canopy leaves is essential for guiding irrigation and fertilization management, diagnosing nutrient deficiencies and stress, predicting yield, and optimizing harvest timing. SPAD value is a relative indicator for indirectly estimating chlorophyll content in leaves, showing a high positive correlation with actual chlorophyll levels and reflecting the health status of plant foliage. This is a core step in realizing intelligent and precise cultivation management of litchi orchards. In recent years, extensive research has been conducted on the SPAD, achieving significant progress overall. However, the relationship between SPAD variation and environmental factors has often been neglected, necessitating further investigation.

The measurement principle of SPAD is based on the selective absorption characteristics of chlorophyll molecules for light at specific wavelengths. By calculating the optical density difference of transmitted light at 650 nm (chlorophyll absorption peak) and 940 nm (near-infrared reference band) through leaves, an empirical relationship model between this difference and chlorophyll concentration is constructed. Due to its advantages of convenient operation, non-destructive testing, and real-time monitoring, this method has become a mainstream technique for in field chlorophyll monitoring. In field crops such as rice and wheat, a mature SPAD–nitrogen–yield correlation system has been established (Kim and Kim, 2024) (Duque et al., 2023). In fruit tree research, crops such as citrus and apple have demonstrated significant correlations between SPAD values and photosynthetic efficiency as well as nutritional status, providing a basis for precision fertilization (Varga et al., 2025). For litchi, research has also made phased progress: scholars have verified a linear positive correlation (R² > 0.8) between SPAD values and laboratory spectrophotometric measurements through destructive sampling, and established basic regression models accounting for varietal differences (Wei et al., 2024); further studies have shown that canopy vertical structure, leaf developmental stage, and phenological phase transitions significantly affect the stability of SPAD values, necessitating stratified sampling or seasonal calibration strategies to improve reliability (Xie et al., 2023). At the same time, texture features extracted from high resolution UAV imagery quantify canopy spatial heterogeneity, which is closely related to canopy structure and within canopy physiological variability. These structural cues can complement pure spectral indices, especially in orchards where crown architecture and background effects vary (Wang et al., 2024).

However, the existing technical approaches still face three main challenges: Firstly, the issue of coverage in rapid detection—while handheld SPAD meters offer high accuracy at single points, they are inadequate for rapidly covering large orchards. On the other hand, although low-altitude drone remote sensing or satellite multispectral imaging can capture large scale multispectral data of litchi canopies, there is a lack of rapid analysis methods, resulting in insufficient accuracy of point to area conversion models (Ji et al., 2023). Secondly, dynamic environmental interference has not been fully modeled. SPAD, as a dynamic physiological indicator, is influenced by environmental parameters such as light intensity, temperature, and humidity fluctuations, which in turn distort reflectance/VI and can also introduce variability in SPAD meter readings (Xiong et al., 2015). While most existing inversion models are based on static assumptions and fail to effectively incorporate environmental parameters. Thirdly, shallow integration of multi-source data—while IoT sensors can collect real-time environmental parameters and multispectral images contain complementary information, current studies mostly employ simple multiple linear regression or traditional machine learning methods for integration, failing to fully explore the nonlinear interactions and temporal dependencies among high dimensional features. These limitations indicate that the dynamic detection methods for SPAD in litchi still require further improvement. Additionally, it is essential to address how to effectively integrate environmental parameter features with SPAD in litchi, analyze their inherent relationships, and provide theoretical references for precision orchard management.

In recent years, the Internet of Things (IoT) has gradually gained widespread application as one of the key technologies in smart agriculture. Currently, IoT is primarily utilized for information collection, equipment control, and environmental monitoring in agricultural settings. Jamshidi et al (Jamshidi et al., 2025). developed an IoT based intelligent monitoring and management system for apple orchards to monitor environmental conditions and manage irrigation and fertilization, yet monitoring of physiological parameters of fruit trees remains relatively insufficient. Imdaad et al (Imdaad et al., 2024). constructed an RFID system based orchard monitoring and management system, in which each tree and fruit was tagged with an RFID label and registered in a cloud database, enabling orchard status visualization and inventory management via a cloud server. These applications preliminarily demonstrate the capability of IoT to enhance the precision of orchard production management. However, there remain challenges in deeply enabling precision management for litchi. First, existing systems generally suffer from data silos, where environmental sensor data lacks effective correlation with plant physiological indicators such as the SPAD. This results in a lack of effective linkage in environment–physiological parameter response models, limiting the in depth application of IoT technology in orchards. Meanwhile, there is still limited research on deep mining of IoT environmental data; multi-source time-series data are mostly used only for threshold alarms or simple regression analysis, failing to incorporate spatiotemporal characteristics to construct growth state prediction models. For perennial crops like litchi, temporal variations across annual growth cycles are key to characterizing their physiological status. In summary, innovative methods that integrate real-time IoT environmental data into multispectral image analysis to enhance the robustness of SPAD inversion are of great significance for improving management in litchi orchards (Rankhambe and Mulajkar, 2025). Existing models based solely on multispectral image analysis still tend to overlook issues such as reflectance distortion caused by abrupt environmental changes and reduced accuracy of spectral inversion models. These shortcomings result in current IoT applications in litchi orchards remaining largely at the data collection level, without forming a closed-loop system of perception–analysis–decision-making.

In the field of non-destructive detection of crop physiological parameters, multispectral imaging and its inversion techniques have been widely applied. The theoretical foundation of using multispectral imaging to invert crop physiological parameters lies in the selective absorption and scattering characteristics of components such as SPAD, water, and nitrogen for photons in different spectral bands (Huang et al., 2021). Huang et al (Huang et al., 2024). designed a transferable model to estimate canopy SPAD across different growth stages and tree species. They utilized a drone equipped with a multispectral imaging device to establish a univariate model based on GNDVI, achieving high prediction performance. On the other hand, image texture features, as a key dimension of spatial domain analysis, can quantify morphological information such as leaf surface roughness and canopy structural complexity through algorithms like the Gray Level Co-occurrence Matrix (GLCM) and Local Binary Patterns (LBP), thereby providing spatial structural compensation for spectral analysis (Wang et al., 2025). Zhao et al (Zhao et al., 2024). adopted a multi-source fusion method combining multispectral and near-infrared spectroscopy to establish a library of various litchi image texture features. They conducted non-destructive assessments of litchi quality and diseases, introduced multiple machine learning models, and achieved rapid non-destructive detection of multiple fruit biological factors through cross fusion methods. Although this study made breakthroughs in inverting crop physiological parameters using multispectral remote sensing image processing, physiological parameters such as SPAD in crops like litchi are correlated with growth cycles and environmental parameters. Relying solely on multispectral images makes it difficult to accurately invert physiological data across all growth stages, as traditional models struggle to capture the relationship between temporal features and multi modal parameters. For instance, litchi canopy leaves exhibit specular reflection under strong light, leading to abnormal fluctuations in near-infrared band reflectance, yet existing models rarely integrate real-time IoT light data for correction. Furthermore, current research on multispectral image processing predominantly relies on single spectral indices and fails to fully utilize texture information in high-resolution images to enhance feature representation. The dynamics of SPAD in litchi depend on complex changes during its growth phenological stages, resulting in reduced accuracy when applied across different scenarios.

In recent years, new technologies represented by machine learning and deep learning have been gradually and widely adopted. Their core value in the intelligent management of orchards lies in automatically uncovering complex mapping relationships between multi-source data and crop physiological states through algorithms (Guimarães et al., 2024). Traditional machine learning methods such as SVM and RF focus on static feature analysis. Yan et al (Yan et al., 2025). established a composite dataset using remote sensing data and onsite measured SPAD data of pear leaves, and introduced machine learning methods including RF and SVM to develop inversion models for pear phenotypic parameters based on spectral reflectance and vegetation index. On the other hand, in terms of the fusion and modeling of multi-source heterogeneous data, many researchers have applied deep learning techniques, such as convolutional neural networks (CNN) and long short-term memory networks (LSTM), to enhance feature extraction capabilities (Research on litchi image detection in orchard using UAV based on improved YOLOv5, 2025). However, existing studies still suffer from weak feature fusion mechanisms. Most models rely solely on data sources such as multispectral images and fail to effectively integrate IoT time-series environmental data, multispectral band features, and high dimensional texture parameters, leading to the loss of critical feature information. Moreover, specialized datasets for litchi are limited in scale, and there are significant structural discrepancies and insufficient volumes across different datasets. These shortcomings hinder the practical application of machine learning and deep learning methods in the accurate estimation of SPAD in litchi, necessitating further research on integrated models tailored to the characteristics of litchi SPAD.

In summary, multi-source fusion combined with a temporal model and feature level attention will better capture time lagged environmental effects and canopy spatial heterogeneity, thereby improving SPAD prediction performance and stability across stages. This study addresses the physiological characteristics of litchi across different phenological stages by constructing an IoT system for detecting litchi growth environmental parameters. The method employs drone based multispectral imaging to capture canopy images of litchi trees, from which spectral reflectance is extracted to establish a dataset of multiple vegetation index. Additionally, textural features of litchi fruit canopy images are thoroughly extracted to create a feature texture dataset. A fusion method integrating physiological parameters of litchi trees is designed and developed. By combining ground synchronized SPAD measurements of litchi leaves, a horizontal comparison is conducted with traditional machine learning methods to validate the advantages of the proposed fusion theory and methodology. This work provides theoretical support for predicting SPAD values in litchi trees and offers a decision making basis for the intelligent management of litchi orchards.

## 2 Materials and methods

### 2.1 Description of the study area

**Figure 1:**
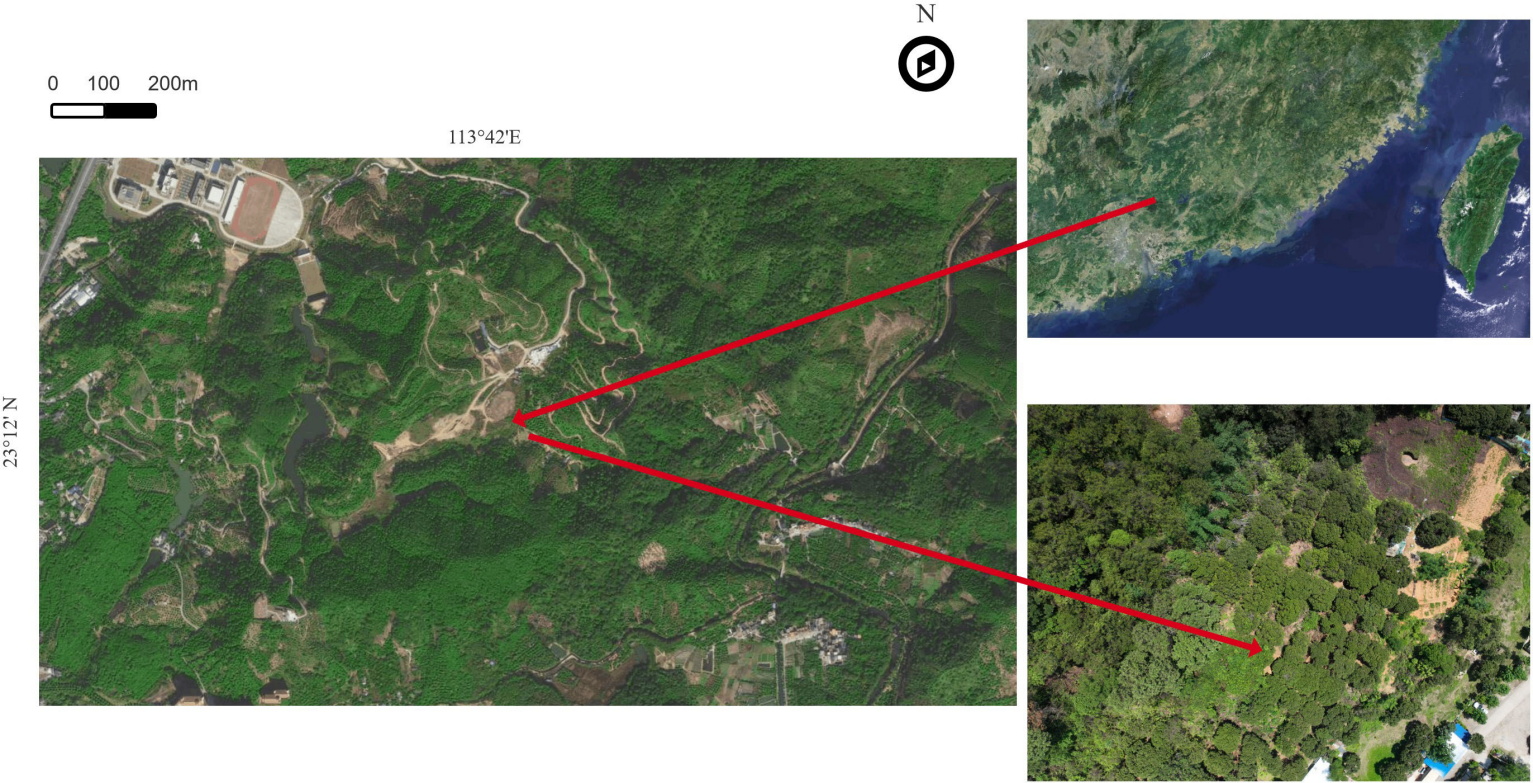
Overview of the study area.

The experimental site is located at the Conghua Litchi Expo Park (113°36′50″E, 23°35′8″N) in Guangzhou, Guangdong Province, China. The orchard covers an area of approximately 20 hectares (about 300 mu), primarily cultivated with perennial and grafted litchi trees. It hosts a total of 116 litchi varieties, with trees averaging about 4 m in height, 8 m in canopy diameter, and spaced approximately 5 m apart. The orchard receives abundant rainfall and demonstrates favorable yield outcomes. The soil type is classified as red soil, and annual irrigation and fertilization inputs remain stable. For this study, an area of approximately 3,000 m² within the orchard was selected. This area features hilly terrain and includes densely planted fruit trees, as shown in **Figure 1**.

### 2.2 Environmental data acquisition via IoT system

This system has constructed an IoT based environmental information acquisition system tailored for litchi orchards. Its core employs an STM32F103RCT6 microprocessor(STMicroelectronics, Italy), which coordinates a distributed sensor network to achieve precise sensing and integrated processing of multidimensional environmental parameters. The sensing layer of the system integrates multiple highprecision sensors to ensure data reliability: a triple function soil sensor (JX-3001, Jingxun Tech, Weihai) continuously monitors dynamic changes in root zone soil temperature (± 0.5 °C), volumetric moisture content (± 1%), and electrical conductivity (± 1%); an integrated weather sensor (TH-03, Jingxun Tech, Weihai) simultaneously captures canopy level air temperature (± 0.3 °C) and relative humidity (± 2%); a wind speed sensor (WS-01, Jingyi Tech, Shanghai) records wind speed fluctuations within the range of 0–3.26 m/s with an accuracy of ±0.3 m/s. This setup is supplemented by a tipping bucket rainfall sensor (JDRK-485, Saien Tech, Jinan) to quantitatively record precipitation events with a resolution of 0.2 mm per tip.

In terms of data transmission, the system employs the CC2630 (Texas Instrument, Texas) ZigBee module to construct a self-organizing wireless sensor network, enabling efficient aggregation and preliminary processing of multi node data. Subsequently, encrypted data are uploaded stably to a remote cloud platform every 30 minutes via the WG-8010-232(Tiantong tech, Beijing) GPRS DTU using the MQTT protocol, ensuring real-time performance and reliability of the data. To guarantee continuous operation in field environments, the system innovatively adopts a hybrid power supply system consisting of a lithium battery (Yingneng tech, Dongguan) and a solar panel DJB-18V10WK (Qianyan tech, Changsha), achieving energy self-sufficiency and optimized management. To enhance data completeness and the reliability of model construction, this study deployed five sets of heterogeneous data acquisition nodes, and the node data were mean aggregated with Pandas framework to provide a solid data foundation for subsequent construction of litchi growth models. Through multi parameter cooperative acquisition and transmission, this system offers effective support for precise orchard monitoring and intelligent decision making.

### 2.3 Acquisition of ground based SPAD measurement data

**Figure 2:**
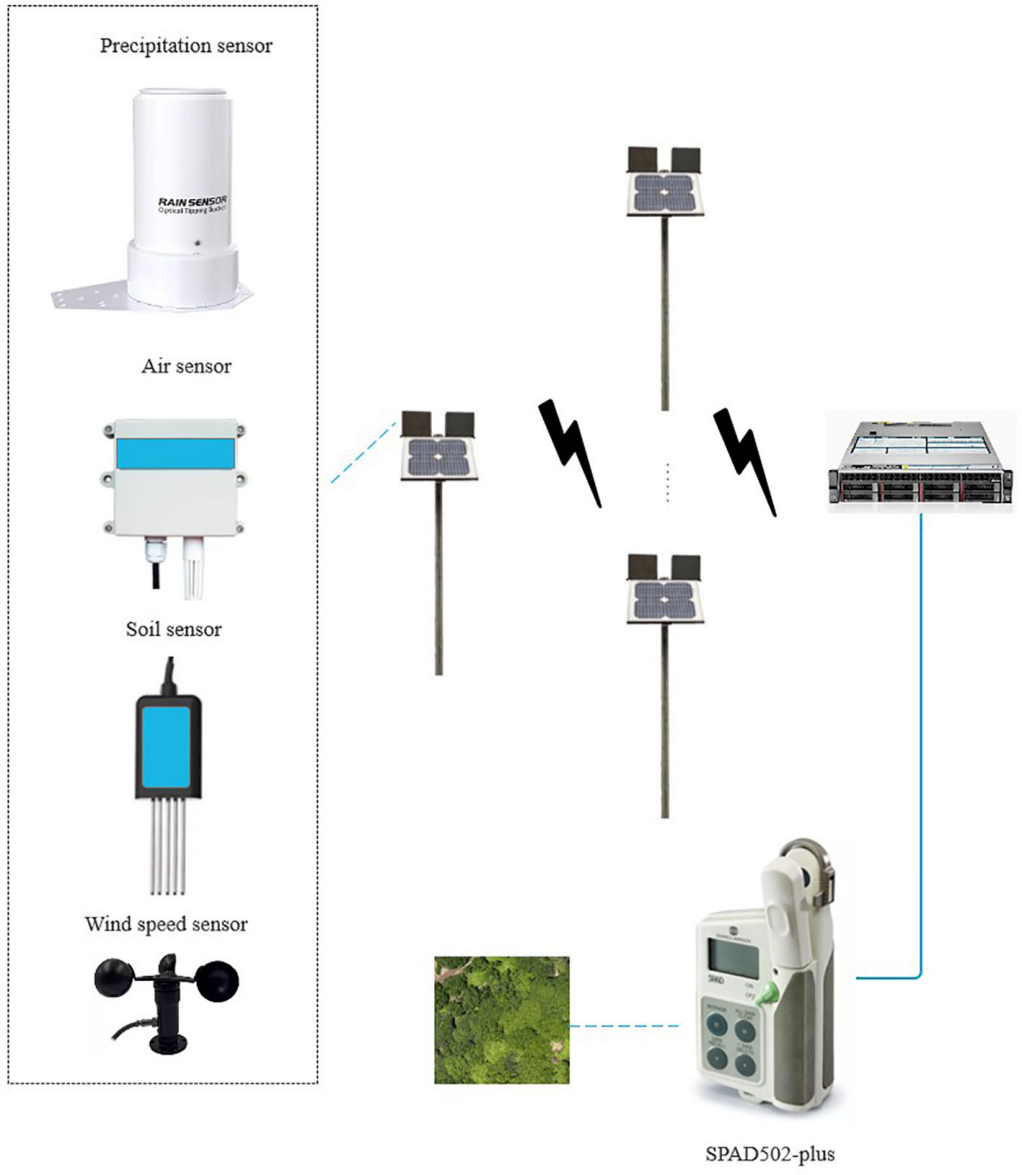
Litchi environmental data acquisition system.

Based on the research area illustrated in **Figure 1**, this study implemented a systematic and continuous monitoring protocol for SPAD across the complete annual growth cycle of litchi (from March 2023 to February 2024). Data collection was conducted using the SPAD-502 Plus (Konica Minolta, Japan), adhering to a baseline sampling frequency of once per week. The sampling design employed a spatial randomization strategy: within each of the four monitored sub-orchards, four sample trees were randomly selected; from each sample tree, five healthy current year functional leaves were uniformly chosen from the canopy, which ensures avoidance of the main leaf vein, visible lesions, and mechanical damage. To avoid interference from direct strong light and dew, as shown in **Figure 2**, measurements were taken using the SPAD-502 Plus SPAD meter between 9:00 and 11:30 am. For each leaf, 5 points were measured along the axis from leaf tip to petiole, and the average SPAD was recorded to ensure the reliability of the data for each leaf. In total, 16 individual litchi trees constituted the biological replicates with repeated and independent measures across the annual cycle. Over the entire year, a total of 5,184 valid data records were obtained. These were synchronously linked with IoT meteorological data (light, temperature, humidity) from corresponding areas to construct a spatiotemporally matched multi-source dataset (Parida et al., 2024). Furthermore, to accurately capture physiological dynamics during key phenological stages such as flower bud differentiation, young fruit expansion, and autumn shoot growth, this study implemented an intensified sampling strategy during these phases, increasing the frequency to twice per week. Based on the systematic monitoring and associated data described above, this study established a high spatiotemporal resolution dedicated database for litchi SPAD. This database not only records the continuous variation of SPAD, but also by correlating environmental factors, provides a solid data foundation for subsequent in depth investigation into the spatiotemporal heterogeneity of litchi leaf physiology under environmental regulation and for constructing related prediction models.

### 2.4 Method for acquiring multispectral data with UAV

**Figure 3:**
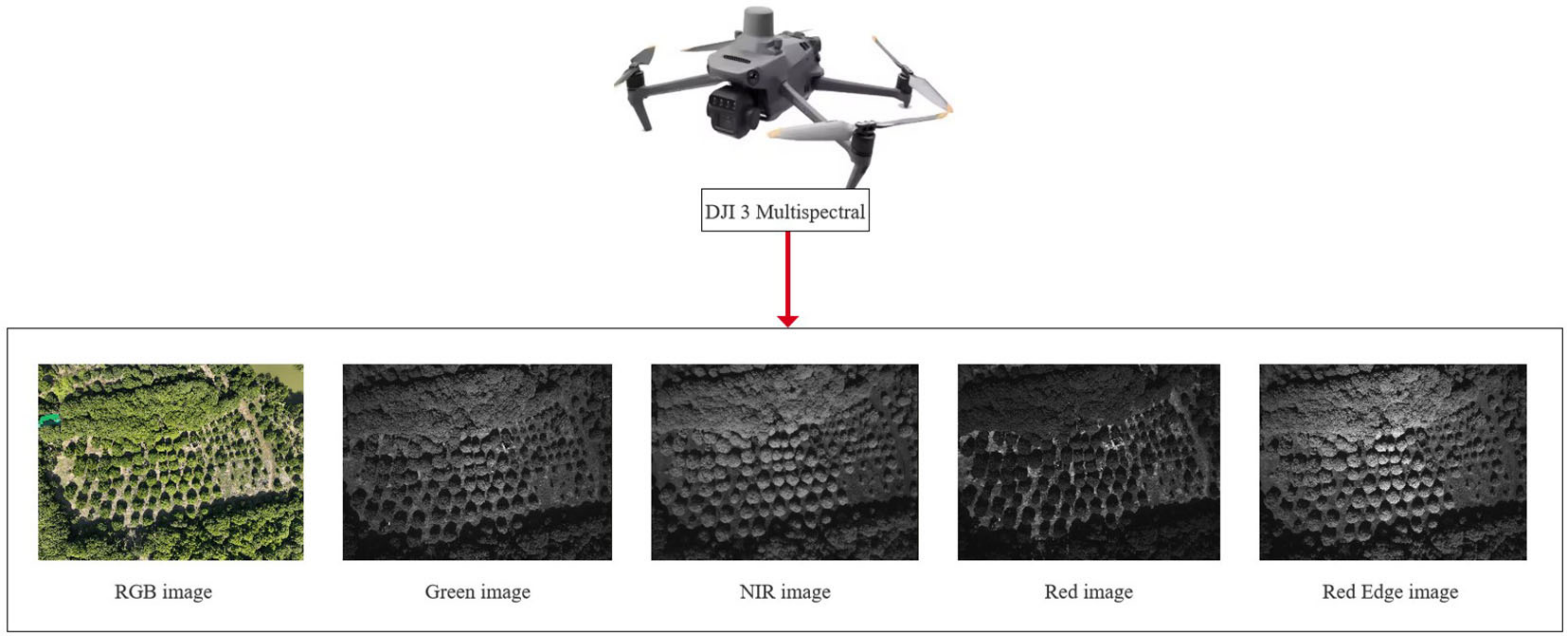
Overview of UAV multispectral data collection.

The multispectral data acquisition for the litchi orchard covered the complete growth cycle from March 2023 to February 2024, utilizing a DJI 3M (Dajiang tech, China) drone equipped with an integrated multispectral imaging system. This system includes one RGB lens and four monochromatic sensors corresponding to the green (560 nm ± 16 nm), red (650 nm ± 16 nm), red edge (730 nm ± 16 nm), and near-infrared (840 nm ± 26 nm) spectral bands, as shown in **Figure 3**. Flight missions were strictly scheduled according to key phenological stages: double monthly surveys during flower bud differentiation, full bloom, young fruit expansion, coloring and ripening, and autumn shoot growth, while single monthly surveys were conducted during non-critical periods. Each flight was performed under clear, cloud free conditions between 10:00 and 14:00 around noon, avoiding extreme weather such as typhoons to prevent operational interference. The flight altitude was fixed at 120 meters, with forward and side overlap rates set at 80% and 70%, respectively. Solar irradiance recorded synchronously by the drone’s built in light sensor was used for radiometric correction. A total of 791 valid images were acquired throughout the year. The raw images from each survey period, combined with solar radiation data from the onboard sensor, were mosaicked to generate 4 band orthophotos. Flight timestamps and corresponding coordinates of SPAD sampling points in each sub-area were recorded simultaneously. Vegetation index values and texture feature values for the regions of interest (ROI) in the litchi canopy were extracted using ENVI 5.6 and spatially linked with ground-based SPAD sampling points from the same period.

### 2.5 Preprocessing of UAV multispectral imagery

**Figure 4:**
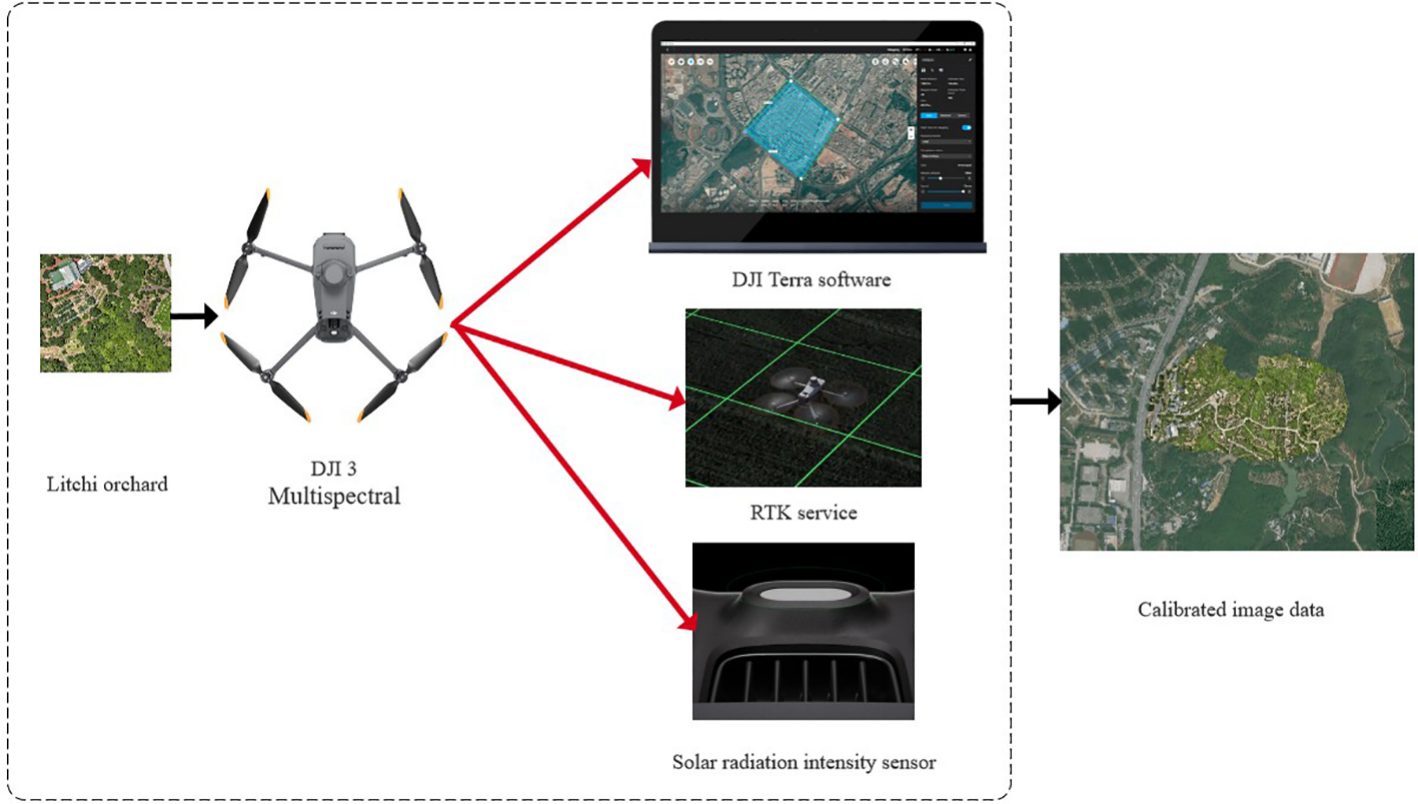
Multispectral image preprocessing workflow.

This study utilized multispectral data acquired by the DJI Matrice 3M drone. As illustrated in **Figure 4**, the DJI Terra (Dajiang tech, China) professional software platform was employed to perform an integrated, end-to-end preprocessing workflow from raw data to high precision surface reflectance data. This workflow comprehensively incorporates solar radiation sensor readings and high precision Real-Time Kinematic (RTK) positioning data, integrating them with multispectral image data to achieve the final synthesis and reconstruction of high precision multi-band spectral imagery (Li et al., 2023). The specific procedure is as follows: after importing the original images into the DJI Terra software, the system first references the spectral response functions embedded in the drone’s multispectral camera and factory provided absolute radiometric calibration parameters to automatically convert the raw digital number (DN) values for each band into radiance at the sensor’s aperture. Subsequently, the software automatically retrieves the solar irradiance data recorded synchronously during the flight and, combined with information such as sensor attitude and solar azimuth/elevation angles, performs top-of-atmosphere radiometric correction based on its built in radiative transfer model, converting the data to surface reflectance. This approach significantly enhances the efficiency of preprocessing large batches of multispectral images (Liu et al., 2024) and ensures a standardized and transparent data preprocessing workflow. In terms of geometric processing of the multispectral images, leveraging the drone’s integrated centimeter level RTK real-time dynamic positioning service provides precise position and attitude information for each image frame. DJI Terra utilizes this high precision POS data along with its automated multi-view image matching and dense matching algorithms to achieve sub-pixel-level automatic registration and orthorectification of multispectral bands. This ensures accurate spatial alignment across multi temporal data, laying a solid foundation for the ground air matching of SPAD data. Ultimately, this process enabled the construction of a high quality dataset integrating IoT data, SPAD data, and multispectral data.

### 2.6 Preprocessing methods for texture features and vegetation index feature calculation

Based on the preprocessed multispectral images acquired throughout the year, nine vegetation index with clear indicative significance for the physiological status of the litchi canopy were systematically extracted to quantify the growth conditions of litchi across different phenological stages (Han et al., 2024). The vegetation index used in this study are listed in **Table 1**. Among them, to assess the fundamental parameters of the litchi canopy structure, three classic vegetation index were selected. The Normalized Difference Vegetation Index (NDVI) effectively reflects changes in the Leaf Area Index, and its annual variation range indicates favorable growth conditions of the litchi canopy. The Green Normalized Difference Vegetation Index (GNDVI) is sensitive to leaf nitrogen content, and its relatively stable observed values suggest minor intra annual fluctuations in the nitrogen status of litchi. The Red Edge Normalized Difference Index (NDRE) demonstrates significant responsiveness to changes in SPAD concentration, especially during critical phenological stages such as flowering and fruit setting. On the other hand, to mitigate interference from orchard soil background, particularly during periods of low vegetation cover or dry seasons, on the extraction of canopy spectral information, two optimized vegetation index were introduced. The Soil Adjusted Vegetation Index (SAVI) effectively reduces the influence of bare soil reflectance on vegetation signals by incorporating a soil adjustment factor, with the correction effect being particularly pronounced during dry seasons. The Modified Soil Adjusted Vegetation Index (MSAVI) further optimizes this approach, significantly improving monitoring accuracy in areas with low canopy closure. Regarding the characterization of SPAD in litchi, relevant indices were calculated. Among them, the Ratio Vegetation Index (RVI) exhibited a strong linear correlation with leaf SPAD, serving as an important reference for leaf photosynthetic capacity. The Difference Vegetation Index (DVI) showed sensitivity to water stress in litchi plants, and specific thresholds can be set to enable early warning of water stress. The Chlorophyll Index (CI) is particularly useful for analyzing SPAD degradation dynamics during leaf senescence. Additionally, the Wide Dynamic Range Vegetation Index (WDRVI) extends the responsiveness to high SPAD values, avoiding the saturation phenomenon commonly observed with conventional indices in high value ranges, thereby enabling more precise monitoring of the physiological status of litchi trees.

**Table 1:** Vegetation index formulas (Li et al., 2025).

VI	Formula
NDVI	(NIR-R)/(NIR+R)
GNDVI	(NIR-G)/(NIR+G)
NDRE	(NIR-RE)/(NIR+RE)
SAVI	1.5×(NIR-R)/(NIR+R+0.5)
MSAVI	2×NIR+1-√((2×NIR+1)²-8×(NIR-R))/2
RVI	NIR/R
DVI	NIR-R
CI	NIR/RE-1
WDRVI	(0.2×NIR-R)/(0.2×NIR+R)

Based on the radiometrically and geometrically corrected multispectral images of the litchi orchard, this study employed the Gray Level Co-occurrence Matrix (GLCM) algorithm to quantitatively analyze the spatial structural characteristics of litchi leaves. By examining the joint distribution of pixel gray values under specific spatial relationships, GLCM effectively captures the periodicity, directionality, and complexity of canopy texture (Liu et al., 2019). As shown in **Table 2**, a total of eight texture indicators sensitive to canopy structure were extracted in this study. Among them, mean and variance respectively characterize the average reflectance intensity within the region of interest and its fluctuation range, reflecting the overall reflectance intensity of the canopy and the heterogeneity of internal patches. Homogeneity describes the uniformity of local image areas, with higher values indicating more uniform texture; contrast quantifies the magnitude of differences between neighboring pixels, with its value positively correlated with the degree of local variation in the image, often used to identify edges or furrow depth. Dissimilarity is similar to contrast but differs in computation, also reflecting pixel differences; entropy measures the randomness and complexity of gray level distribution in the image, with higher entropy indicating more complex texture; angular second moment (also known as energy) reflects the orderliness of the image’s gray level distribution, with larger values indicating more regular texture patterns; correlation assesses the linear dependency of pixel gray values in a specified direction. In this study, the computation of texture features was batch processed using ENVI 5.6 software and its relevant plugins. First, raster layers for the eight texture features described above were generated for each of the four spectral bands. Subsequently, pure canopy regions were accurately extracted through visual interpretation combined with pre delineated canopy boundaries. Finally, for each litchi canopy ROI, texture feature values were statistically summarized across scales, ultimately constructing a multi dimensional texture feature matrix for subsequent analysis. This matrix provides essential data support for a deeper understanding of the variation patterns in litchi canopy structure across phenological stages.

**Table 2:** Texture features formulas (Li et al., 2023).

Texture features	Formula
MEA	$\mu_{i}=i\sum_{ij=0}^{N-1}P_{ij};\mu_{j}=j\sum_{ij=0}^{N-1}P_{ij}$
VAR	$\sigma_{i}^{2}=\sum_{i,j=0}^{N-1}P_{ij}{\left(i-\mu_{i}\right)}^{2};\sigma_{j}^{2}=\sum_{i,j=0}^{N-1}P_{ij}{\left(j-\mu_{j}\right)}^{2}$
HOM	$\sum_{i,j=0}^{N-1}\frac{P_{i,j}}{1+{\left(i-j\right)}^{2}}$
CON	$\sum_{i,j=0}^{N-1}P_{i,j}{\left(i-j\right)}^{2}$
DIS	$\sum_{i,j=0}^{N-1}P_{i,j}|i-j|$
ENT	$\sum_{i,j=0}^{N-1}P_{i,j}(-\text{ln}P_{i,j})$
SEC	$\sum_{i,j=0}^{N-1}P_{i,j}$
COR	$\sum_{i,j=0}^{N-1}P_{i,j}\frac{\left(i-\mu_{i})(j-\mu_{j}\right)}{1+{\left(i-j\right)}^{2}}$

### 2.7 Feature set construction and data preprocessing

In the construction and preprocessing of the feature set, this study integrates three types of heterogeneous data—IoT environmental time-series data, texture features extracted from multispectral images, and vegetation index to form a high dimensional composite feature matrix with spatiotemporal consistency, which serves as input for subsequent model training and testing (Hurlbert, 1984). To address the differences in units and scales among the data, a Min-Max normalization method is first applied to scale all features, expressed by the Equation 1:

(1) $$X_{\text{norm }}=\frac{X-X_{min}}{X_{max}-X_{min}}$$

where $X_{\text{norm }}$ is the normalized data and X is the original data. $X_{min}$ and $X_{max}$ represent the maximum and minimum of the original data, respectively. After processing, the value range of the dataset is uniformly scaled to the interval [0, 1]. The feature vector constructed in this study spans a complete annual growth cycle from March 2023 to February 2024. It is based on ground SPAD sampling points collected at a frequency of once to twice per week during key phenological periods. Since the original IoT data were collected at hourly intervals, this study employed the mean aggregation method from the pandas scientific computing package to aggregate hourly environmental data into daily values, thereby generating daily environmental feature data. For temporal alignment with the UAV multispectral image texture feature data, this study used the flight dates and their corresponding litchi key phenological stages as core observation timestamps. This approach established a link between IoT environmental data and SPAD data, creating heterogeneous data fusion anchors driven by phenological milestone points. To address issues such as irregular SPAD data collection intervals and the misalignment between SPAD data, IoT data, and multispectral texture features—while also considering that SPAD changes slowly without abrupt shifts or significant fluctuations due to meteorological factors—this study anchored the data to UAV flight dates. Linear interpolation was applied to estimate SPAD values on the flight dates using IoT environmental data and observed SPAD measurements. Approximately 30% of SPAD records were derived via linear interpolation to align with UAV flight dates. This method achieved temporal alignment among IoT data, multispectral image texture features, and SPAD data. Building on this, the study successfully integrated data of varying dimensions and structures based on a unified temporal scale, forming a comprehensive dataset that incorporates litchi growth phenology and diverse environmental parameters. This ensures robust data support for subsequent model training and inference.

### 2.8 Construction of MLSTM SPAD prediction model with attention mechanism

**Figure 5:**
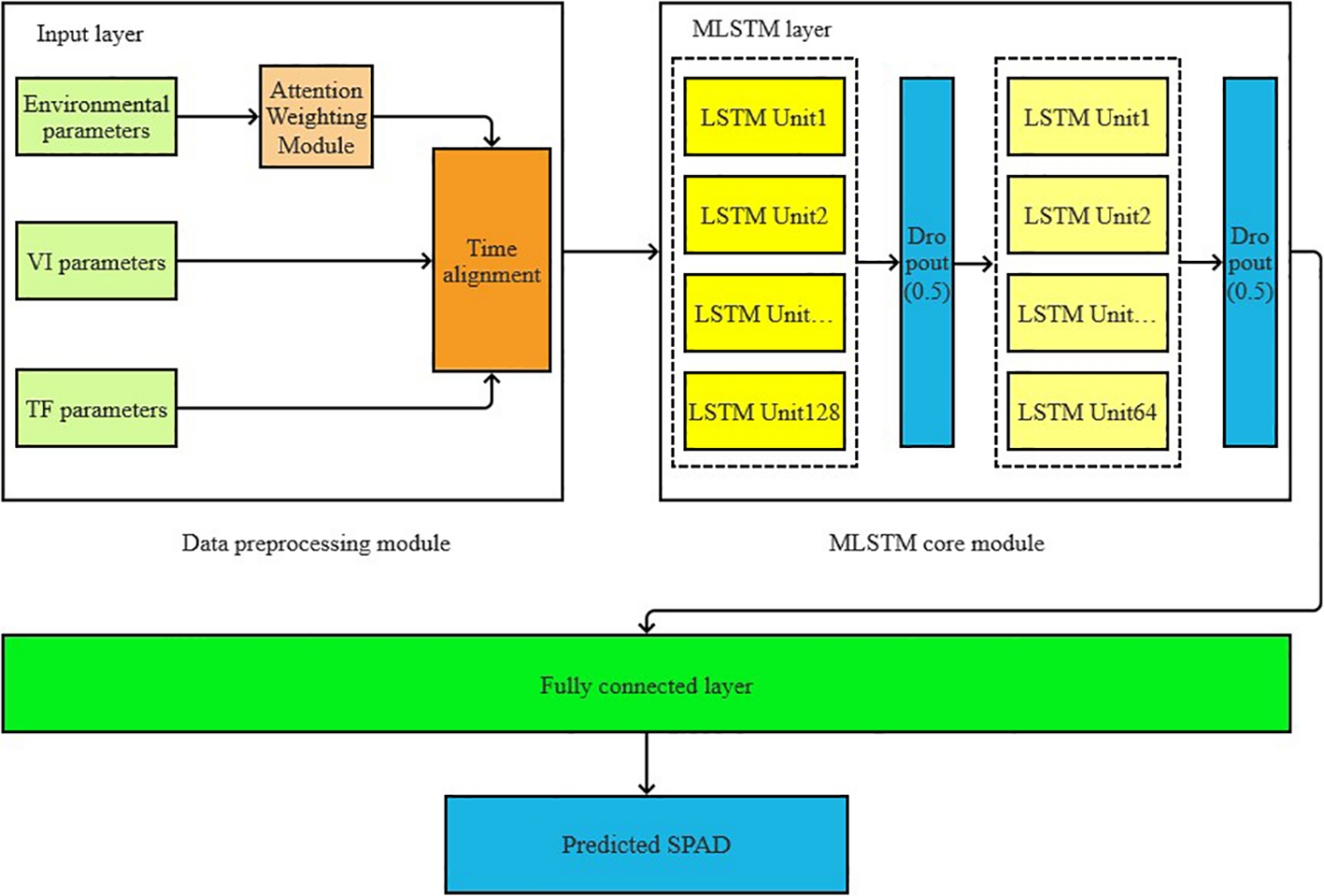
Overall model architecture.

To accurately estimate the SPAD of litchi leaves and overcome the limitations of traditional models in fusing high dimensional, heterogeneous multi-source data—such as insufficient mining of feature interactions and a lack of adaptive focus on key texture information—this study constructs an attention based bidirectional long short-term memory network model, characterized by a many-to-one temporal feature pattern. This paper proposes a model that deeply integrates IoT environmental time-series data, UAV-based multispectral vegetation index, and high-dimensional texture features. By introducing an attention mechanism to dynamically enhance canopy texture feature patterns critical for SPAD prediction, and utilizing MLSTM to capture long and short-term spatiotemporal dependencies among features, the model achieves high precision estimation of SPAD. The overall architecture of the model is shown in **Figure 5**, whose core is a multi branch deep neural network. The input layer receives three types of heterogeneous features: 1) Environmental time-series features: IoT environmental data from litchi orchard sensors and their statistical derivatives; 2) Vegetation index features: nine vegetation index; and 3) Texture features: 32 dimensional texture features. To improve feature processing efficiency, the texture feature is first normalized, then fed into independent feature level attention weighting module. The processed features, after temporal alignment, are jointly input into a multivariate LSTM layer for deep spatiotemporal feature extraction and fusion. Finally, the abstract features are integrated through fully connected layers to output the final predicted SPAD value.

**Figure 6:**
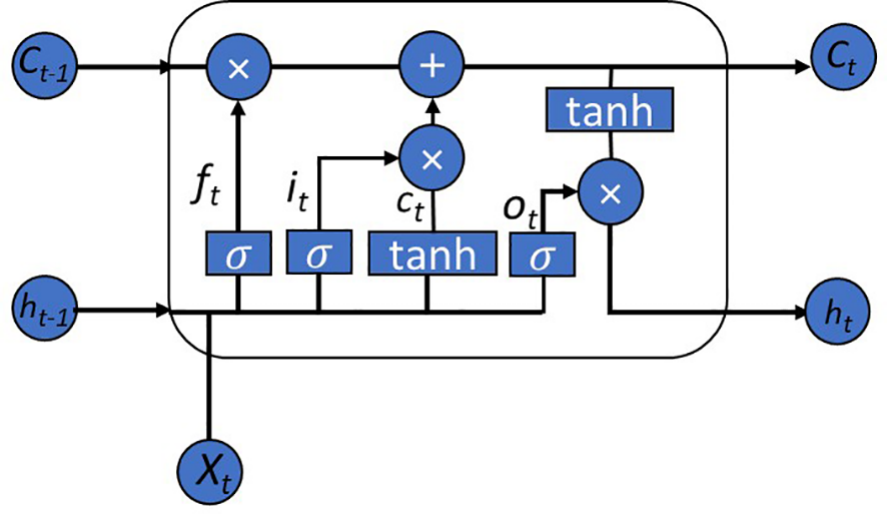
LSTM unit structure diagram.

In terms of constructing time-series training input data, the core idea of the MLSTM proposed in this paper is to utilize historical feature data from N consecutive days prior to the target SPAD measurement date to predict the SPAD value on that day. That is, for the *t* target prediction day, the feature data from days *t*-N (N was set to 7 days) are used as model inputs. For feature alignment, regarding vegetation index and texture features extracted from UAV images, if flight data are unavailable on a specific day, linear interpolation based on data from adjacent days is applied to fill the gaps, ensuring sequence continuity. Using a sliding window method, multiple sequence samples of length 7 are generated for each tree. Ultimately, the entire dataset is restructured into a three-dimensional tensor. Furthermore, to address high dimensional feature redundancy and noise interference, and to enable the model to autonomously focus on feature patterns most relevant to leaf physiological status, this study designs a feature level attention mechanism. This mechanism learns a dynamic weight vector to rescale the importance of the original features. For the *t* sample, its texture feature vector can be represented as ${\text{x}}_{tex}^{\left(t\right)}\in {\mathbb{R}}^{D}$, where D represents feature dimensions in this dataset. Firstly, attention weight coefficients are computed through a learnable fully connected layer and a Sigmoid nonlinear activation function, expressed by the Equations 2, 3:

(2) $$z_{t}=W_{a}\cdot x_{tex}^{\left(t\right)}+b_{a}$$

(3) $$a_{t}=\sigma (z_{t})$$

where ${\text{W}}_{a}\in {\mathbb{R}}^{D\times D}$ ${\text{x}}_{a}\in {\mathbb{R}}^{D}$ represent training parameters, σ is the sigmoid function, which reweights each feature dimension to characterize the relative importance of each feature dimension. Then, this paper employs the Hadamard product to perform element wise multiplication for weighting the original features, with the formula as follows (Equation 4):

(4) $${\tilde{x}}_{tex}^{\left(t\right)}=a_{t}⨀x_{tex}^{\left(t\right)}$$

where ${\tilde{x}}_{tex}^{\left(t\right)}$ represent the features of average weighted, of which role is to enhance the feature indicators more closely related to SPAD while suppressing irrelevant redundant feature information, thereby improving the model’s interpretability. As shown in **Figure 5**, the attention weighted features are concatenated along the time dimension to form the fused feature sequence, which can be expressed as Equation 5:

(5) $$X_{t}=[x_{env}^{\left(t\right)};{\tilde{x}}_{vi}^{\left(t\right)};{\tilde{x}}_{tex}^{\left(t\right)}]$$

where ${\text{x}}_{env}^{\left(t\right)},\;{\text{x}}_{vi}^{\left(t\right)}and\;{\tilde{\text{x}}}_{tex}^{\left(t\right)}$ denote environmental features, index features, and texture features, respectively, which will be input to the MLSTM network. As shown in **Figure 6**, LSTM effectively captures long and short-term dependencies in time series through gating mechanisms, which include a forget gate $f_{t}$ to control the amount of input information, an input gate $i_{t}$, a temporary cell state $c_{t}$, a hidden layer state $h_{t}$, and an output gate $o_{t}$. Its input is $x_{t}$, and is Equations 6–11:

(6) $$f_{t}=\sigma (W_{f}\cdot [h_{t-1},x_{t}]+b_{f})$$

(7) $$i_{t}=\sigma (W_{i}\cdot [h_{t-1},x_{t}]+b_{i})$$

(8) $$c_{t}=\tanh(W_{c}\cdot [h_{t-1},x_{t}]+b_{c})$$

(9) $$C_{t}=f_{t}*C_{t-1}+i_{t}*c_{t}$$

(10) $$o_{t}=\sigma (W_{o}[h_{t-1},x_{t}]+b_{o})$$

(11) $$h_{t}=o_{t}*\tanh(C_{t})$$

where Equation 6 is the update formula for the forget gate, whose value range is (0, 1), determining the amount of information to be discarded. σ transforms the output of the forget gate. $W_{f}$ and $b_{f}$ are the weight and bias parameters of the forget gate, respectively. $h_{t-1}$ and $x_{t}$ represent the previous hidden state and the current input value, respectively.**Equation 7** is the update formula for the input gate, which controls the amount of information allowed to enter the LSTM cell. $W_{i}$ and $b_{i}$ are the weight and bias of the input gate, respectively. When the value of $i_{t}$is 1, it indicates that the input data are permitted to pass through the inputgate. **Equations 8**, **9** are the update formulas for the candidate cell state and the cell state, respectively. They integrate the previous hidden state and the current input value. $W_{c}$ and $b_{c}$ are the weight and bias of the candidate cell state, respectively. The update of the cell state $C_{t}$ is jointly determined by the forget gate, input gate, candidate cell state, and the previous cell state. Specifically, $f_{t}*C_{t-1}$ represents the information to be discarded from the previous cell state, while $i_{t}*c_{t}$ represents the amount of information to be updated in the current cell state. In**Equation 10** $o_{t}$ is the output of the output gate, with $W_{o}$ and $b_{o}$ being the weight and bias of the output gate, respectively. It is determined by the previous hidden state and the current input value. Under the action of the activation function, the value range of the cell state $C_{t}$ is limited at (0, 1), and together with the output gate value, it determines the current hidden state value.

### 2.9 Machine learning models for comparative performance in SPAD prediction

#### 2.9.1 Random forest model

To establish a robust baseline model and compare it with the MLSTM framework proposed in this study, a targeted design and systematic hyperparameter tuning were applied to the Random Forest (RF) model. Random Forest was selected as the baseline representing traditional machine learning methods due to its strong adaptability to high dimensional, nonlinear data and its inherent interpretability. Considering the characteristics of the multi-source heterogeneous feature set in this study, the standard Random Forest was adapted accordingly. To address potential scale differences and nonlinear interactions among features, all input features underwent the same data preprocessing steps before training. During the training process, the n_estimators parameter, which controls the number of trees in the forest, was set to 100 to balance model accuracy and computational efficiency. Simultaneously, the max_depth parameter was set to 20 to prevent overfitting to complex noisy data. The min_samples_split and min_samples_leaf parameters, which define the minimum number of samples required to split an internal node and the minimum number of samples required to be at a leaf node, were set to 5 and 2, respectively. This configuration helps control tree structure growth and enhances the model’s overall generalization capability. Furthermore, the max_features parameter was set to sqrt, employing the square root of the total number of features as the strategy for selecting the maximum number of features considered for splitting a node. This approach effectively increases diversity among the trees when handling high dimensional data, thereby maximizing the model’s performance potential.

#### 2.9.2 LightGBM model

To further facilitate a horizontal comparison, this study also introduced the LightGBM model and implemented targeted architectural adaptation along with systematic hyperparameter optimization. LightGBM was selected as a comparative model representing advanced ensemble learning methods due to its high efficiency in handling high dimensional features. For the multi-source heterogeneous feature set constructed in this study, LightGBM’s inherent strengths were fully utilized for adaptation. First, all continuous features were normalized, while for texture features, the full feature matrix was used to address potential multicollinearity among different features, ensuring input feature independence and preventing model weight distortion. Regarding model hyperparameter optimization and training strategies, to fully leverage LightGBM’s potential and ensure its effectiveness as a strong baseline, hyperparameter settings were optimized. The learning rate was set to 0.05 to balance convergence speed and accuracy. Meanwhile, the initial number of trees n_estimators was set to 1000, and early stopping rounds early_stopping_rounds were set to 50. During training, if the validation metric did not improve for 50 consecutive iterations, the training process automatically terminated and returned the model with the best iteration. Through this approach, the optimal number of trees was dynamically determined to be 100, effectively mitigating overfitting while improving training speed. The maximum tree depth max_depth was set to 8 to control model complexity and avoid excessive sensitivity to noise during training. To enhance model robustness, the minimum number of samples in a leaf min_data_in_leaf was set to 10. The feature sampling ratio feature_fraction and data sampling ratio bagging_fraction were set to 0.8 and 0.9, respectively. These parameters randomly select subsets of features and data for training in each iteration, serving as key mechanisms in LightGBM to implement row/column sampling, increase diversity among trees, improve model generalization, and accelerate training, thereby laying the foundation for enhanced model performance.

### 2.10 Model training, evaluation, and dataset partitioning

Regarding model training evaluation and dataset partitioning, to avoid future information leakage, a time ordered splitting strategy was adopted: data from the early to middle growth period of the 2023 season—comprising the first 80% of the time span for all sample trees—were used to construct the training set, while data from the final 20% of the growth period were strictly reserved as an unseen test set, thereby simulating a realistic scenario for SPAD value prediction. In the partitioned datasets, the training set primarily includes stages including bud differentiation, full bloom, young fruit expansion, and early fruit development, while the test set mainly covers fruit ripening and harvest, postharvest autumn shoot growth, and winter dormancy-phenological stages that never appear in training dataset. By constructing a cross−phenological dataset, the model’s generalization capability can be enhanced. To examine the distribution of different features, Pearson correlation analysis was performed to assess the relationships between individual features and SPAD values. On this basis, to enable a fair comparison among models, all models in this study—including RF, LightGBM, MLSTM, and the standard LSTM—were optimized using the same training and test sets. Among them, to allow traditional models to also leverage temporal information, the constructed historical feature sequences were flattened into a very long static vector as their input, while the LSTM based models directly received the raw sequential data for training. This partitioning simulates the process of operational forecasting. During training, 5−fold time−series cross−validation (i.e., the rolling−origin method) was employed for hyperparameter tuning, where earlier data blocks were used for training and subsequent blocks for validation, while strictly preserving temporal order. Furthermore, to deeply analyze the performance sources of the LSTM, ablation experiments were conducted by training the LSTM separately using only environmental sequences, only vegetation index sequences, only texture sequences, and finally the full feature sequences. In terms of model implementation, Python along with intelligent computing packages such as scikit-learn were employed for model construction and testing (Yu et al., 2025). For model evaluation, Root Mean Square Error (RMSE) and the coefficient of determination (R²) were used to characterize the interpretability of the fitting results. Their Equations 12, 13 are as follows:

(12) $$\text{RMSE}=\sqrt{\frac{\sum_{m=1}^{N}{\left(y_{\text{pred}}-y_{\text{cal}}\right)}^{2}}{N}}$$

(13) $$R^{2}=1-\frac{\sum_{m=1}^{N}{\left(y_{\text{pred }}-\bar{y_{\text{cal }}}\right)}^{2}}{\sum_{m=1}^{N}{\left(y_{\text{cal }}-\bar{y_{\text{cal}}}\right)}^{2}}$$

where $y_{\text{pred}}$ is the predicted value, $y_{\text{cal}}$ is the measured value, $\bar{y_{\text{cal }}}$ is the mean value of the measured value and N represents the number of samples.

## 3 Results

### 3.1 Correlation analysis results

**Figure 7:**
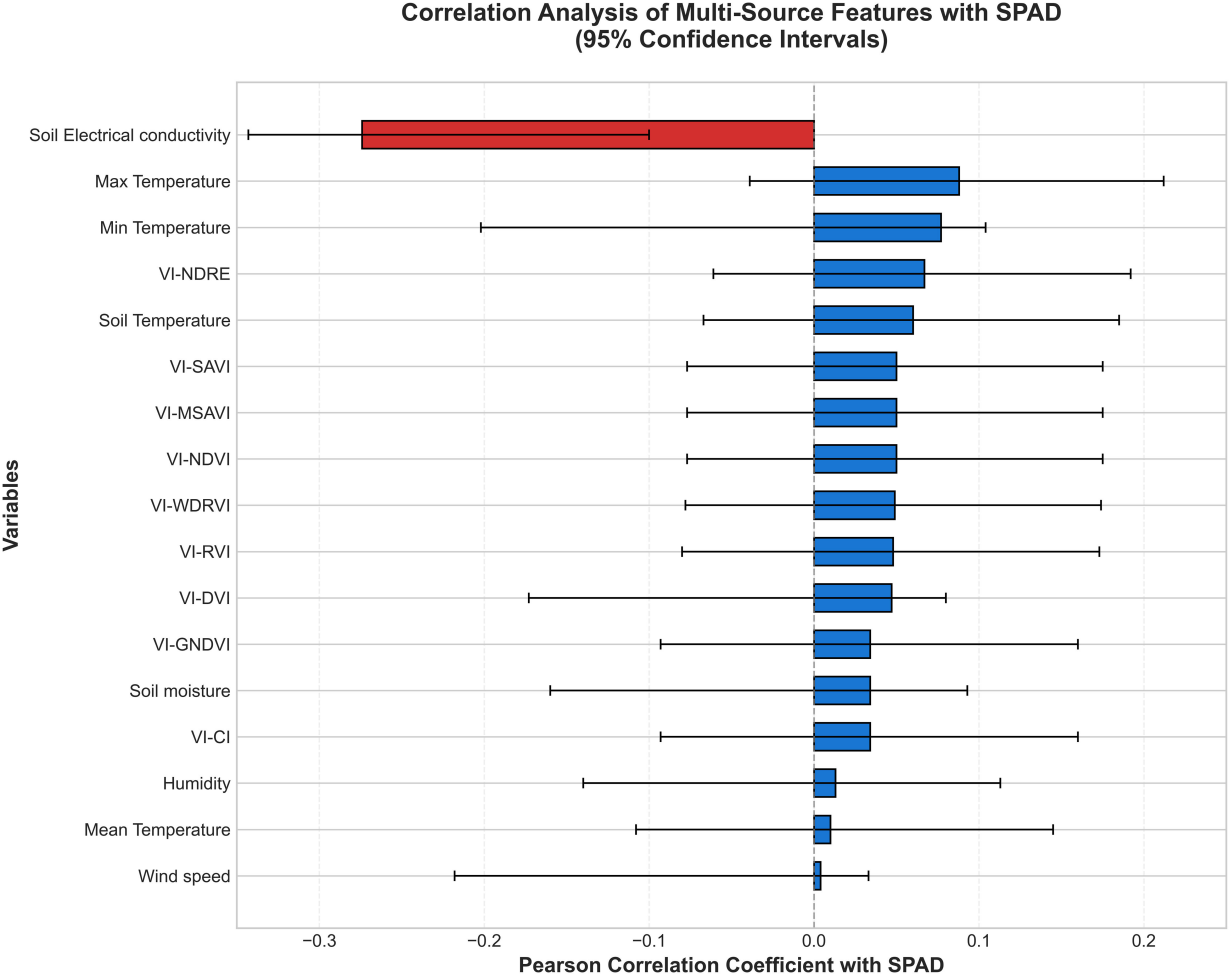
Correlation analysis results of multi-source features with SPAD values in litchi.

**Figure 7** presents the correlation analysis results between multi-source IoT monitoring data, remote sensing vegetation index, and leaf SPAD values in the litchi orchard. The values in parentheses following each indicator represent the 95% confidence interval. The analysis shows that soil electrical conductivity, soil temperature, and most vegetation index exhibit positive correlations with SPAD values. Among these, vegetation index VI-NDRE and VI-NDVI demonstrate relatively higher correlations, indicating that canopy spectral information serves as a good indicator of leaf SPAD. In contrast, meteorological factors such as maximum temperature, minimum temperature, average temperature, and wind speed show low correlation coefficients with SPAD values, with some displaying weak negative correlations. These results suggested that individual meteorological factors had a limited direct influence during the observation period. Overall, vegetation indices and soil related parameters exhibited stronger correlations with SPAD values compared to meteorological factors.

**Figure 8:**
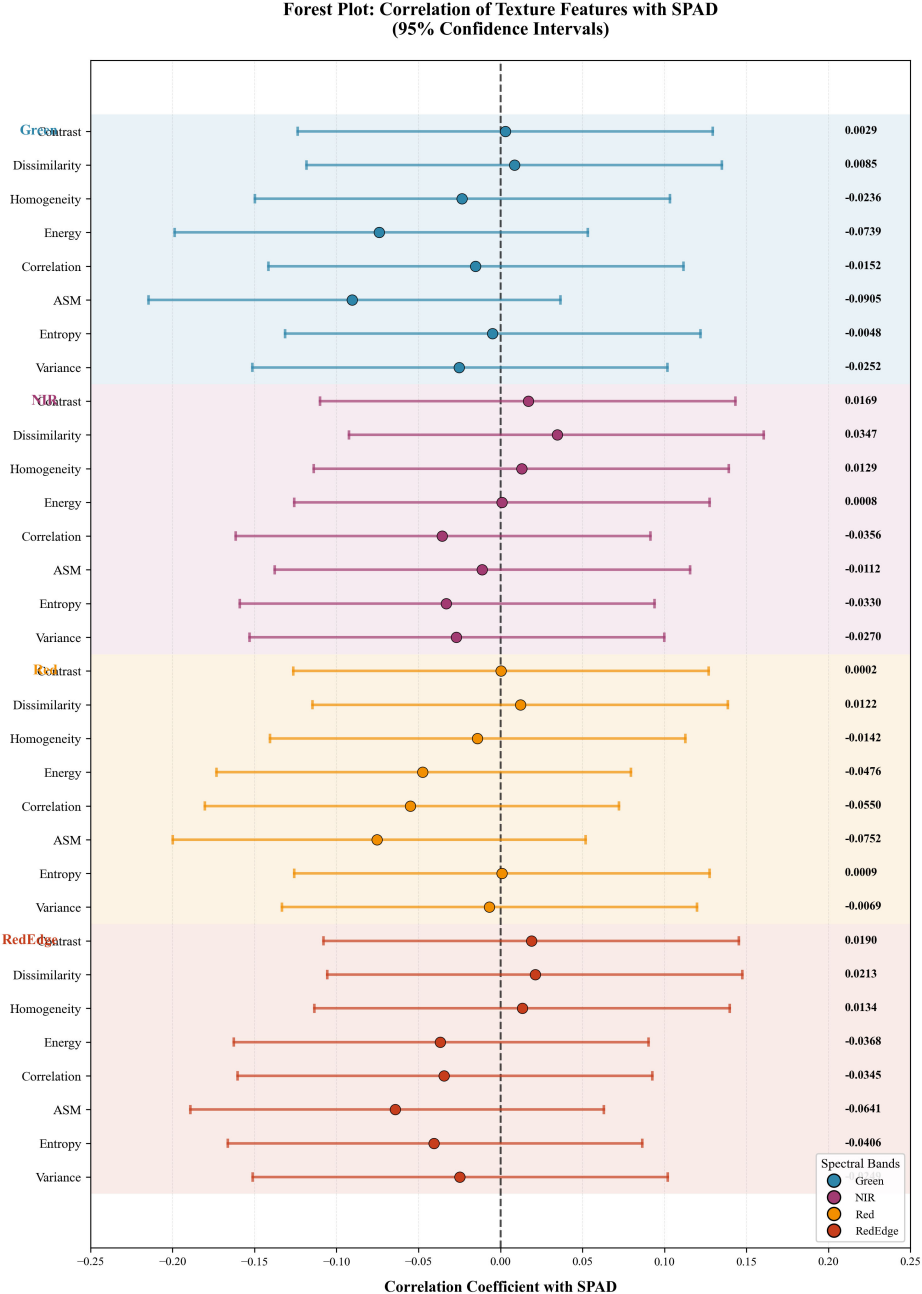
Correlation analysis results between texture features and SPAD values.

**Figure 8** illustrates the correlations between image−derived texture features and SPAD values across different spectral bands. The results indicate clear differences in the response of texture features to SPAD values among the bands. In the green and red bands, the correlations between various texture features and SPAD values are generally weak. In contrast, in the near−infrared (NIR) and red−edge (RedEdge) bands, certain texture features exhibit relatively pronounced positive correlations, with the contrast feature in the red−edge band showing the highest correlation coefficient. This suggested that texture information derived from the NIR and red−edge bands possesses a certain potential for characterizing the spatial distribution of chlorophyll in the litchi canopy, and can serve as effective feature inputs for subsequent remote−sensing inversion models of SPAD.

### 3.2 Correlation analysis comparison of prediction performance of different models for litchi SPAD value

**Figure 9:**
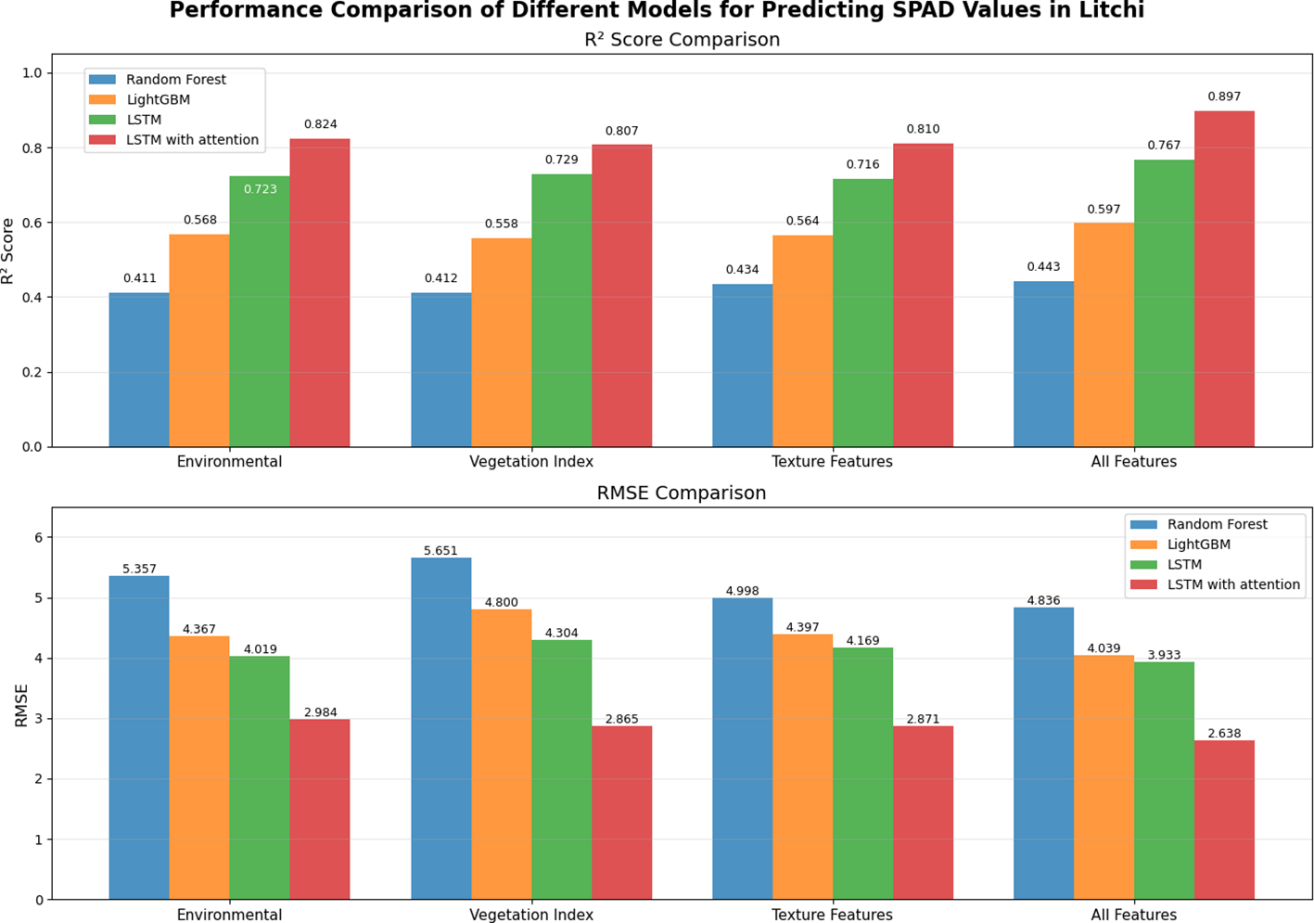
Model performance comparison results.

This study evaluated the prediction performance of the proposed model and comparative models for SPAD values of litchi leaves under four feature combinations. The coefficient of determination (R²) and root mean square error (RMSE) between measured and predicted values are shown in **Figure 9**. From the R² comparison in **Figure 9**, it can be observed that prediction performance exhibits a stepwise improvement trend as model complexity and feature number increase. Traditional machine learning models, RF and LightGBM, demonstrate moderate predictive capability: the R² values of RF range from 0.411 to 0.443, while LightGBM performs slightly better, with R² values ranging from 0.558 to 0.597. In contrast, the use of the LSTM model in this study leads to further performance enhancement, with its R² values increasing to a range of 0.716 to 0.767, indicating that LSTM can better capture complex temporal and nonlinear relationships in the data. After incorporating the weighted attention mechanism, the improved MLSTM model significantly outperforms all other models across all feature combinations. Particularly when all features are used, the R² reaches 0.897, meaning that the model can explain nearly 90% of the variation in SPAD values. This improvement in accuracy is likely attributed to the attention mechanism’s ability to enhance the model’s focus on key features or temporal information.

In terms of the RMSE comparison, it can be observed that the RF model exhibits the largest prediction error, with RMSE values ranging from 4.836 to 5.651, while both the LightGBM and the standard LSTM models show reduced errors. After incorporating the weighted attention mechanism, the MLSTM model achieves the lowest prediction error under all conditions. Particularly when using the full feature combination, the RMSE decreases to 2.638, representing a reduction of 34.7% compared to the best performing traditional model, LightGBM. This demonstrates the superior predictive performance of the improved model. Paired t-tests are conducted on absolute prediction errors, where MLSTM vs RF p<0.001, vs LightGBM p<0.001, vs LSTM p=0.003. The test results confirmed significance at p<0.01 for all pairwise comparisons.

Regarding the impact of feature combinations, the “All features” combination consistently yields the best performance for nearly all models, which underscores the importance of multi−source data fusion: environmental factors, spectral vegetation index, and image texture features provide complementary information for the accurate estimation of SPAD values. For the LSTM model with attention mechanism, although a single feature set can already achieve a relatively high R², its performance is further improved to 0.897 when all features are fused. This synergistic enhancement effect is also evident in other models, demonstrating the effectiveness of the methodology proposed in this study.

### 3.3 Analysis of prediction results of different models for litchi SPAD

**Figure 10:**
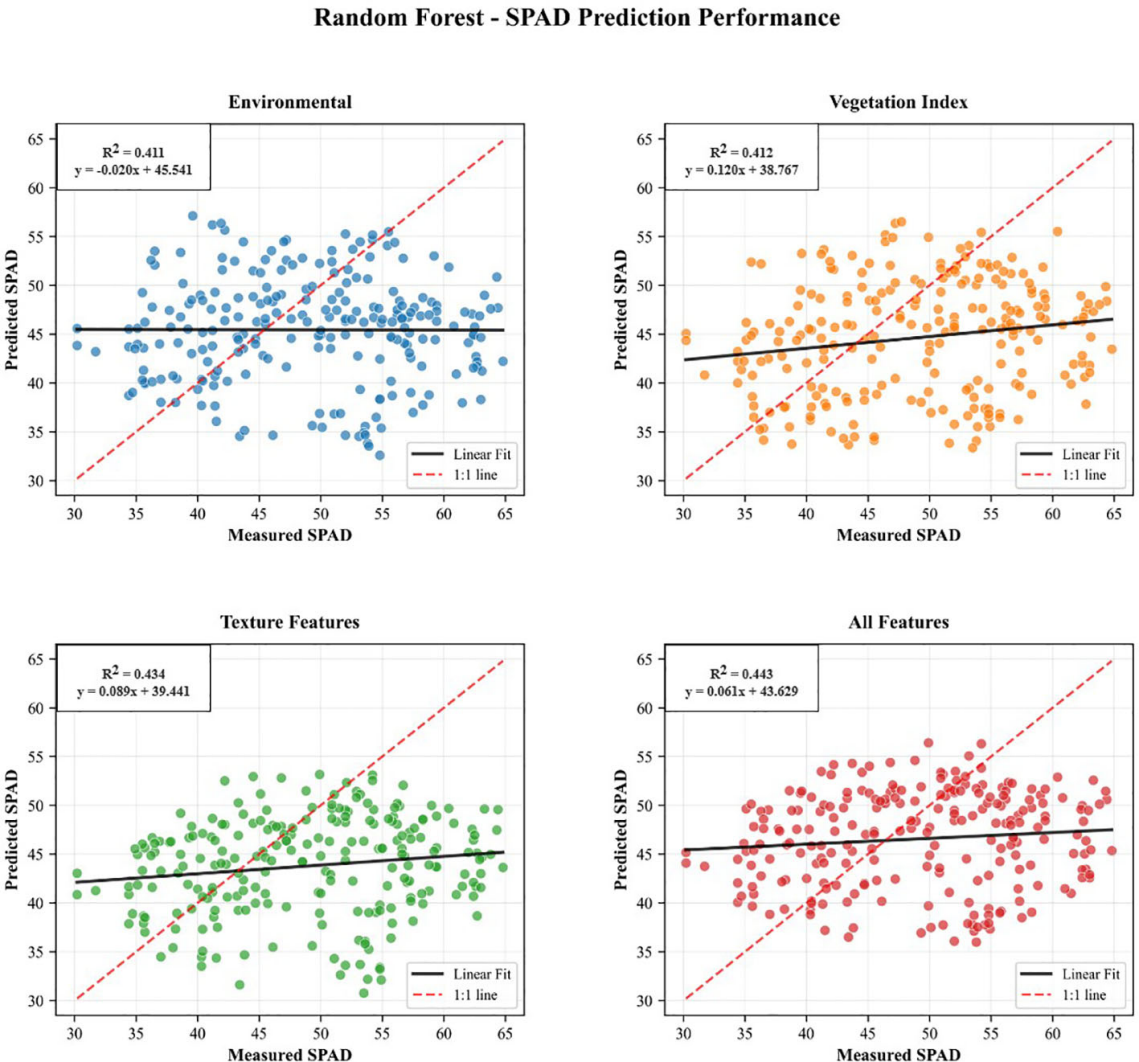
Comparison of fitting performance between measured and predicted SPAD values under the RF model.

**Figure 11:**
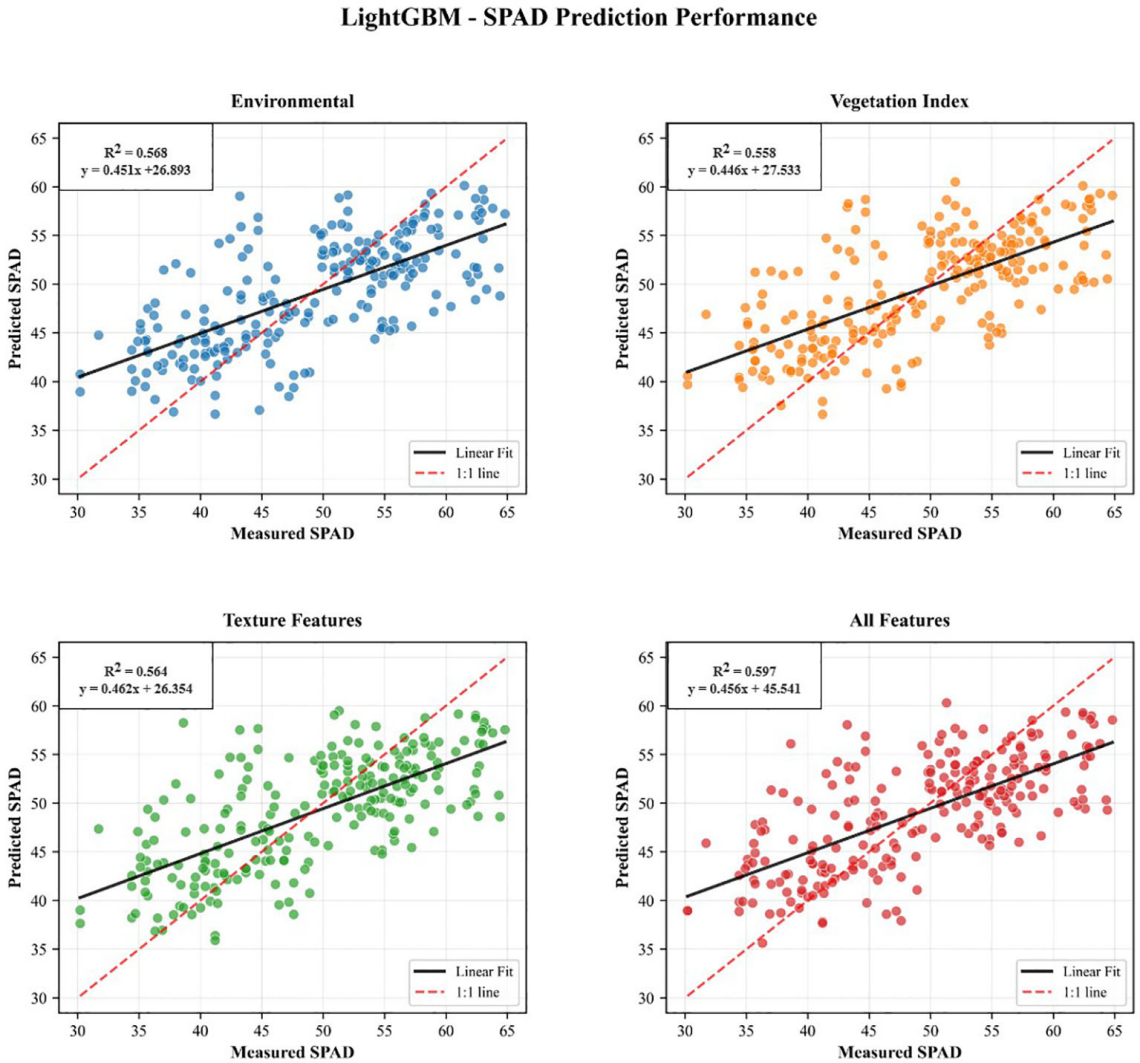
Comparison of fitting performance between measured and predicted SPAD values under the LightGBM model.

**Figure 12:**
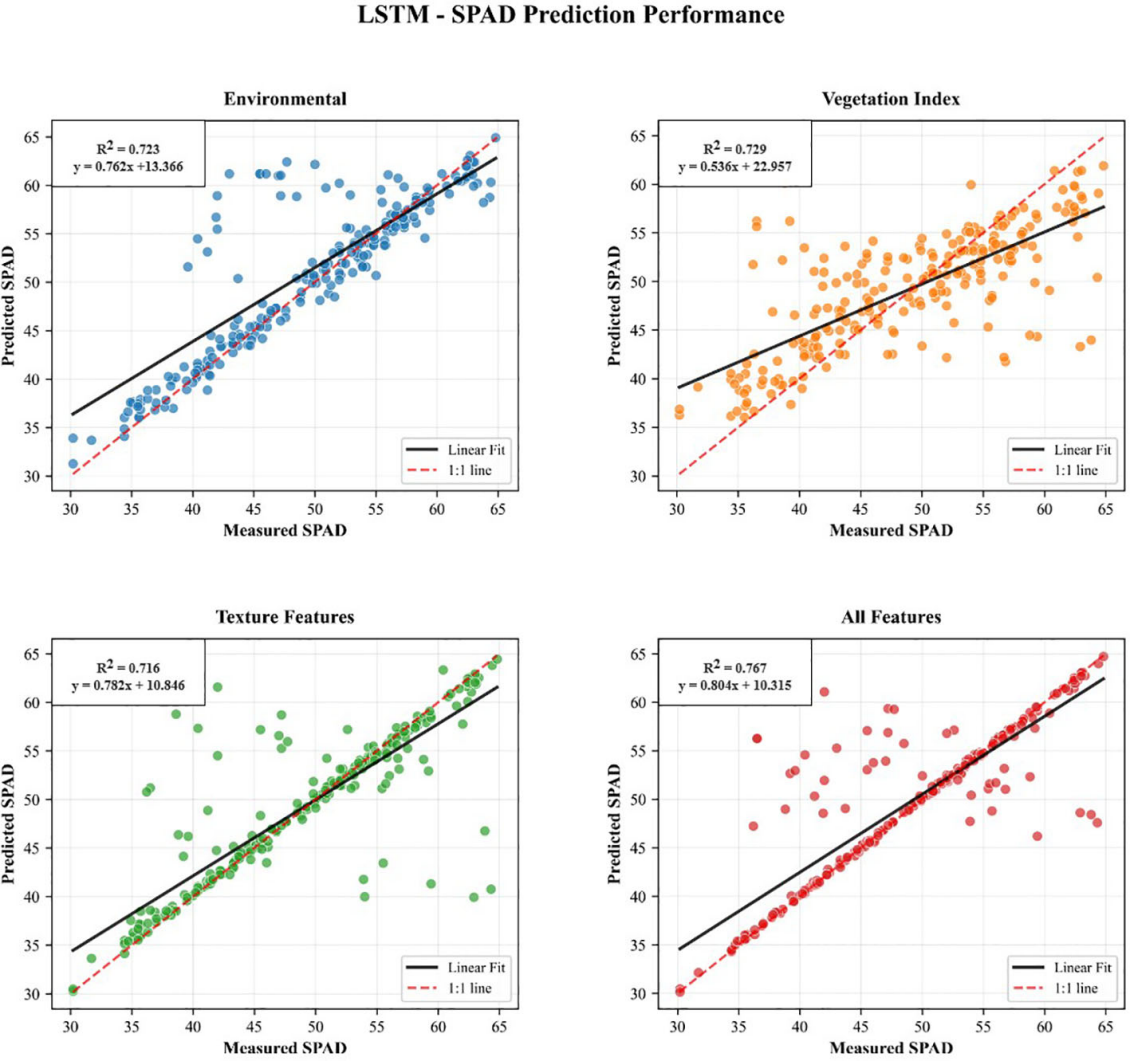
Comparison of fitting performance between measured and predicted values of SPAD under the LSTM model.

**Figure 13:**
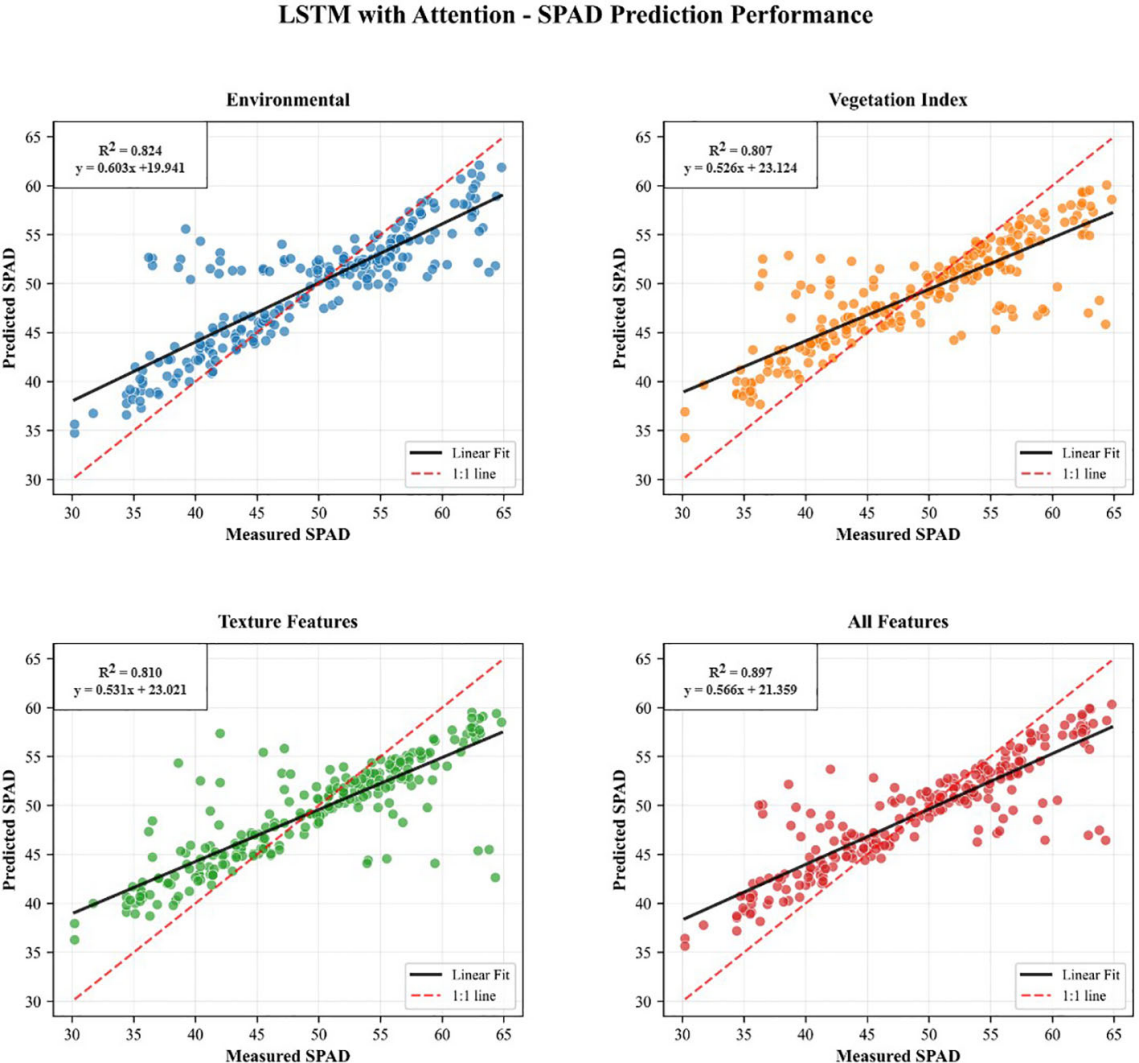
Comparison of fitting performance between measured and predicted SPAD values under the MLSTM model incorporating weighted attention mechanism.

Based on the fitting analysis between measured and predicted SPAD values, this study systematically evaluated the prediction performance of the four models, with the results shown in **Figures 10**-**13**. The Random Forest (RF) model performed poorly across all feature combinations, revealing its limitations in handling such complex regression tasks. Under the four input conditions—”environmental features,” “vegetation index,” “texture features,” and “all features”—the R² values of the RF model ranged only from 0.411 to 0.443. Notably, when using only “environmental features,” the slope of the fitted line approached zero (y = −0.020x + 45.541), indicating that the model barely captured any effective variation patterns related to SPAD values from the environmental variables.

In contrast, the LightGBM model, based on a gradient−boosting framework, demonstrated stronger learning capability. As shown in the “environmental features” subplot of **Figure 11**, under the same feature input, its R² improved to 0.568, and the predicted versus measured values exhibited a clear positive correlation trend (y = 0.451x + 26.893). However, the data points were more scattered in the high−value region, suggesting systematic bias in the model—tending to underestimate high SPAD values and overestimate low SPAD values. This result implies that although LightGBM outperforms RF, the information provided by a single type of feature (here, environmental features) remains insufficient, limiting the model’s final accuracy.

The introduction of LSTM for processing time-series data and SPAD prediction in this study led to improved performance. Taking the prediction results under the “environmental features” set as an example, as shown in **Figure 12**, the R² value of the LSTM model further increased to 0.723. Its fitting equation (y = 0.762x + 13.366) has a slope closer to 1 and a reduced intercept, indicating a significant improvement in the deviation between predicted and measured values, with data points more tightly distributed along the 1:1 line. Notably, the LSTM model also achieved excellent results when processing texture features. This suggests that its sequence modeling capability may be particularly adept at mining the spatiotemporal structural information embedded within texture features, primarily by capturing seasonal and phenological progression encoded in environmental cues. This process reveals strong instantaneous causal relationships, thereby enabling more accurate inversion than traditional models. Our analysis is based on a dataset collected from a single orchard over one complete annual cycle. While this provides a consistent temporal framework, the generalizability of the findings to other orchards, growing seasons, or cultivars requires external validation in future work—a limitation that is explicitly emphasized in the concluding discussion. Methodologically, the key conclusions presented here are derived from predictions on the held out temporal period, not from in sample performance, thereby focusing on the model’s forecasting capability within the observed cycle.

When the proposed MLSTM model with an integrated attention mechanism was employed for SPAD prediction, its results highlighted the crucial role of adaptive feature selection and fusion. As shown in **Figure 13**, the MLSTM model achieved near optimal fitting performance under the “all features” input, with an R² as high as 0.897. More remarkably, this model maintained highly robust and excellent performance even with single feature inputs, forming a sharp contrast to other models whose performance fluctuated depending on the features used. This outcome demonstrates that the integrated attention mechanism significantly enhances the model’s efficiency in information extraction and fusion by dynamically weighting the importance of different features. Across various feature combinations, the model can autonomously focus on the most relevant information, thereby stably and efficiently approximating the true SPAD values. The fitted line (y = 0.566x + 21.359) and the high density band of data points further confirm the high consistency and low dispersion of the predictions. We also performed a residual analysis of the prediction equation. The Shapiro-Wilk normality test yielded a p-value of 0.12, and the mean residual was -0.31, indicating no strong evidence of systematic association between residuals and fitted values or time order. A sensitivity analysis of the look-back window N showed that performance stabilizes when N = 7 offering the best trade-off in terms of scenario complexity.

## 4 Discussion

### 4.1 Model discussion

This study systematically evaluated the performance of four models with varying complexities in estimating SPAD values of litchi leaves by integrating multi-source heterogeneous data. The results indicate a significant positive correlation between model architecture evolution and prediction accuracy. From traditional machine learning to deep sequence models, and further to the improved model (MLSTM) incorporating feature level attention mechanisms, the predictive capability of the models demonstrated a stepwise enhancement. This phenomenon reveals the complex physiological mechanisms underlying the dynamic changes in SPAD values, which are influenced not only by instantaneous environmental conditions but are also closely related to historical growth states and canopy spatial structure. While traditional RF and LightGBM models can handle high dimensional static features, their “flattened” input processing approach struggles to effectively capture the temporal dynamics within the data, resulting in a performance bottleneck. In contrast, the LSTM model, with its inherent gated recurrent units, can memorize and utilize historical information, thereby more effectively modeling the continuous variation of SPAD values throughout the phenological process. MLSTM uses a windowed sequence, enabling it to learn delayed responses and smooth phenological trajectories. In an orchard over an annual cycle, environmental variables also act as proxies for phenological stage, which can yield predictive power even when same day linear correlation is weak. The proposed MLSTM model with an integrated attention mechanism achieves adaptive weighted fusion of multi-source features. Not only did this model attain near optimal fitting performance under the “all features” input, but more importantly, it maintained highly robust and excellent performance with any single feature set. This proves that the attention mechanism can dynamically focus on the feature dimensions most relevant to the current prediction, significantly enhancing the model’s information extraction efficiency and robustness.

This study breaks through the limitation of relying solely on spectral information prevalent in most existing research. It innovatively integrates IoT based real-time environmental time-series data with vegetation index and texture features extracted from UAV multispectral imagery, constructing a spatiotemporally aligned multi dimensional dataset. This provides a data foundation for addressing the insufficient accuracy of “point-to-area” conversion models and mitigating interference from dynamic environmental factors. At the model level, to overcome the limitation of existing fusion models often being shallow linear models, this study introduces an LSTM architecture capable of capturing temporal dependencies and further incorporates an attention mechanism. Compared with the static models such as RF and SVM used by Yan et al (Yan et al., 2025). for inverting pear tree SPAD values, the proposed model better reflects the continuous physiological processes of crop growth. In contrast to the multi-source fusion method adopted by Zhao et al (Zhao et al., 2024). for litchi quality detection, the attention mechanism introduced in this study enables adaptive selection at the feature level, rather than simple feature stacking, thereby enhancing both model interpretability and accuracy.

### 4.2 Model application and limitations

The MLSTM based SPAD estimation model with an integrated attention mechanism developed in this study provides a directly applicable decision−support tool for precision management in litchi orchards. The high accuracy and low error of the model imply that farmers or orchard management systems can achieve spatialized and near−real−time monitoring of SPAD across large−scale litchi orchards, using readily available IoT environmental data and periodic UAV multispectral imagery. This offers significant practical value: first, in nutrient management, the rapid, large−area SPAD prediction model can provide an intuitive overview of crop growth within the orchard, thereby supplying data support for variable−rate fertilization and helping to avoid resource waste and non−point source pollution. Second, in stress diagnosis, abnormal fluctuations in SPAD values often serve as early signals of water stress or pest/disease infestation; this model enables early warning, allowing timely intervention. Finally, in yield and quality prediction, SPAD is closely related to photosynthetic efficiency and fruit quality. Continuous monitoring of SPAD during key phenological stages can contribute to more accurate yield forecasting and fruit quality assessment. Despite the promising accuracy of the proposed fusion model for SPAD estimation, its practical application faces several limitations and challenges, including potential domain shifts across different orchards and years due to data from a single growing season, inherent constraints in data acquisition such as dependence on UAV flight schedules and stable illumination, reliability issues like signal noise and drift in soil sensors, as well as considerations of operational cost and system maintenance complexity; therefore, to facilitate real world adoption, we recommend establishing robust sensor quality control and optimized flight scheduling prior to deployment, strongly advocate for external validation across diverse years and locations, and suggest that future work focus on building more spatiotemporally diverse datasets and exploring model lightweighting for edge computing deployment to achieve cost effective and robust orchard monitoring.

Although this study achieved satisfactory prediction performance, certain limitations remain and should be addressed in future research. First, the spatiotemporal scope of the dataset. Data collection was confined to a single orchard over one complete annual growth cycle. While this ensured data continuity and consistency, the model’s generalizability across different regions, cultivars, management practices, and abnormal climate years still requires validation. Therefore, broader data collection efforts are needed in the future. Second, the dimensionality of feature extraction. This study primarily utilized second−order texture features extracted via GLCM, which proved effective. However, higher−order texture features or more complex spatial structural features may contain additional valuable information, warranting exploration of more diverse feature extraction methods. Third, model lightweighting and deployment. Although the proposed MLSTM model with attention achieves high accuracy, its computational complexity is relatively high, posing challenges for deployment on portable embedded devices. Developing system−level models that balance accuracy and efficiency is crucial for promoting the practical adoption of this technology. Given the time-series nature of our data, conventional significance tests that assume independent and identically distributed errors are prone to misinterpretation. Therefore, in lieu of such direct comparisons, we have strengthened the current analysis by focusing on the interpretation of practical effect sizes and by demonstrating the consistent pattern of improvement across various feature combinations and models. Further research equipped with multi orchard or multi year datasets, would be well positioned to employ more appropriate dependence aware uncertainty estimation methods, such as block resampling or time-series cross validation, to provide formal statistical inference. Secondly, during the data preprocessing stage, approximately 30% of the SPAD values in the dataset were filled using linear interpolation, based on the assumption of a linear growth pattern in SPAD readings. In a strict evaluation using only field−measured test data, the model demonstrated robust performance. While we acknowledge that including interpolated points in the assessment might slightly improve the reported metrics, we conducted experiments such as cross−validation and significance tests, which indirectly verified the performance advantage of our model. Moving forward, we also plan to consider higher−frequency ground−UAV synchronous measurements in follow−up studies to further enrich the dataset.

## 5 Conclusions

To address the challenge of rapid and accurate estimation of SPAD in large−scale litchi orchards, this study systematically established a multi−source data framework integrating IoT time−series environmental data, UAV−based multispectral vegetation index, and canopy texture features. An innovative prediction model—a Long Short−Term Memory network integrated with a feature−level attention mechanism (MLSTM)—was proposed. Through comparative experiments with traditional machine learning models (RF, LightGBM) and a standard LSTM model, the following main conclusions are drawn:

1. Multi−source feature fusion plays a key role in improving SPAD estimation accuracy. The results demonstrate that integrating environmental factors, vegetation index, and texture features provides complementary information for SPAD estimation. Compared to any single feature set, using the “all−features” combination achieved the best or near−best predictive performance across all models (e.g., the R² of the MLSTM model increased from approximately 0.81 with a single feature set to 0.897), effectively overcoming the limitations of incomplete information from a single data source.
2. Model architecture is a core factor determining prediction accuracy. The results indicate that deep learning models (LSTM, MLSTM), due to their strong temporal modeling capability, significantly outperform traditional machine learning models. In particular, the introduced attention mechanism enables the MLSTM model to adaptively focus on key features, leading to a notable improvement in prediction accuracy (R² = 0.897) and robustness, with RMSE reduced to 2.638.
3. The attention mechanism effectively enhances feature utilization efficiency and model interpretability. The feature−level attention module in the MLSTM model dynamically learns the importance weights of different texture features. This not only optimizes model performance but also provides insights into the intrinsic relationship between canopy spatial structure features and SPAD values, thereby improving model interpretability.

The methodologies and technical framework developed in this study hold clear practical value for enabling intelligent and precision management of litchi orchards. The high−accuracy SPAD estimation model can be used to generate spatial chlorophyll distribution maps, guide variable−rate fertilization, diagnose nutritional stress, and ultimately achieve the goals of improving quality and efficiency while reducing environmental pollution. Although the expected outcomes were achieved in this study, future research could be expanded in the following directions: First, expanding the geographical scope and temporal span of the dataset to validate the model’s generalizability; second, exploring the integration of multimodal data such as hyperspectral and LiDAR to further enhance estimation precision; and third, investigating model lightweighting techniques to promote deployment on edge computing devices, enabling real−time monitoring and decision−making, thereby providing robust theoretical support and practical case studies for the advancement of smart agriculture. Second, exploring dimensionality reduction techniques such as Principal Component Analysis (PCA) to reduce input variable redundancy when integrating environmental, spectral, and textural features, potentially improving computational efficiency without sacrificing predictive accuracy.

## Data Availability

The datasets presented in this study can be found in online repositories. The names of the repository/repositories and accession number(s) can be found below: https://zenodo.org/records/18308090.
